# Tenth International Foamy Virus Conference 2014–Achievements and Perspectives

**DOI:** 10.3390/v7041651

**Published:** 2015-03-31

**Authors:** Magdalena Materniak, Piotr Kubiś, Marzena Rola–Łuszczak, Arifa S. Khan, Florence Buseyne, Dirk Lindemann, Martin Löchelt, Jacek Kuźmak

**Affiliations:** 1 Department of Biochemistry, National Veterinary Research Institute, 24-100 Pulawy, Poland; E-Mails: kubip@piwet.pulawy.pl (P.K.); mrolka@piwet.pulawy.pl (M.R.-L.); jkuzmak@piwet.pulawy.pl (J.K.); 2 Laboratory of Retroviruses, Division of Viral Products, OVRR, CBER, U.S. Food and Drug Administration, Silver Spring, MD 20993, USA; E-Mail: arifa.khan@fda.hhs.gov; 3 Unité d'épidémiologie et Physiopathologie des Virus Oncogènes, Institut Pasteur, 75015 Paris, France; E-Mail: florence.buseyne@pasteur.fr; 4 Institute of Virology, Medical Faculty “Carl Gustav Carus”, Technische Universität Dresden, 01307 Dresden, Germany; E-Mail: Dirk.Lindemann@tu-dresden.de; 5 Department of Genome Modifications and Carcinogenesis, German Cancer Research Center, 69121 Heidelberg, Germany; E-Mail: m.loechelt@dkfz.de

**Keywords:** foamy viruses, infection, zoonotic potential, restriction factors, replication, assembly, FV vectors

## Abstract

For the past two decades, scientists from around the world, working on different aspects of foamy virus (FV) research, have gathered in different research institutions almost every two years to present their recent results in formal talks, to discuss their ongoing studies informally, and to initiate fruitful collaborations. In this report we review the 2014 anniversary conference to share the meeting summary with the virology community and hope to arouse interest by other researchers to join this exciting field. The topics covered included epidemiology, virus molecular biology, and immunology of FV infection in non-human primates, cattle, and humans with zoonotic FV infections, as well as recent findings on endogenous FVs. Several topics focused on virus replication and interactions between viral and cellular proteins. Use of FV in biomedical research was highlighted with presentations on using FV vectors for gene therapy and FV proteins as scaffold for vaccine antigen presentation. On behalf of the FV community, this report also includes a short tribute to commemorate Prof. Axel Rethwilm, one of the leading experts in the field of retrovirology and foamy viruses, who passed away 29 July 2014.

## 1. Introduction

The series of foamy virus (FV) conferences, organized biennially since 1994, have been a unique and important international forum devoted to progress and achievements in FV research. Such meetings have provided attendees an opportunity for scientific exchange of recent data and ideas as well as discussions on all aspects of FVs including FV infections of natural hosts and zoonosis, molecular biology, vector development and host-virus interactions. The International Foamy Virus Conference, held on 24–25 June 2014 in Pulawy, Poland, marked its 10th anniversary, and was organized by the National Veterinary Research Institute (NVRI) and the Committee of Veterinary Sciences Polish Academy of Sciences. The two-day meeting held at the NVRI conference facilities brought together experts and members of their groups to discuss FVs, as well as to experience great Polish hospitality and to enjoy the beautiful Vistula river valley with its gems of Polish culture and history. The meeting provided a state-of-the-art overview of the latest findings and covered the following topics: (1) epidemiology of FVs infections in primates, horses, and cattle, and zoonotic aspects of FVs; (2) FV restriction and immunity; (3) FVs replication, proteins, and assembly; and (4) FVs vectors for gene transfer. 

Topics and themes of the plenary talks and oral presentations are provided below to share knowledge from the FV conference with others in the scientific community and encourage researchers to notice FV topics and join us at the 11th International Foamy Virus Conference, which is being planned to be held in Paris, France in 2016. For further reading we strongly suggest a recent and comprehensive overview of all relevant aspects of FV biology published in a Special Issue on FVs in *Viruses* [1]. 

## 2. Summary of Scientific Sessions

### 2.1. FV Infection and Zoonotic Aspects 

**Arifa S. Khan** (Food and Drug Administration, Silver Spring, MD, USA) opened the session by introducing the audience to FV infections, which are endemic in many species all over the world, and presented data from her laboratory regarding the co-infection of simian FV (SFV) and simian immunodeficiency virus (SIV) in primates. Her presentation summarized the existing knowledge about exogenous and endogenous FVs, experimental animal models to study the replication of FVs, and the influence of naturally occurring SFV infection on SIV disease progression in the *Macaca mulatta* (rhesus macaque**)** model. She highlighted the finding of endogenous sequences in coelacanth, zebrafish, platyfish, cod, slots, aye-aye and one recently described FV of the small insectivore *Chrysocloris asiatica* (Cape golden mole), which provides strong evidence that FVs were already present in the most recent common ancestor of all placental mammals over 100 million years ago [2,3,4]. A. Khan summarized various laboratory models used for *in vivo* studies of the biology of exogenous FVs, including small (rabbits, mice, hamsters) and large animal models (cats, cattle and sheep, monkeys), as well as highlighted the possibility of recombination between FVs of Old World primates, as was observed in the isolates SFVcpz, SFVmcy-2 and SFVmac-R289hybAGM, similar to the situation with the two different feline FV (FFV) serotypes [5]. A. Khan further presented the results of her laboratory’s published study on the influence of naturally occurring SFV on SIV disease progression in the rhesus macaque model [6]. In brief, comparative analysis of SFV+/SIV+ and SFV−/SIV+ monkey groups indicated statistically significant differences in the plasma viral load of SIV between 6–28 weeks, particularly after reaching plateau at 20–28 weeks, in the CD4+ and CD8+ T-cell numbers over the entire study period (2–43 weeks), and in the survival rates evaluated at 49 weeks. There was an increase in the plasma viral load, a decreasing trend in the CD4+ T cells, and a greater number of animal deaths in the SFV+/SIV+ group. The results, although based upon a small number of animals, indicated that pre-existing SFV infection can influence SIV infection and disease outcome in the rhesus macaque model. The study highlighted consideration of the SFV status in evaluating results from SIV pathogenesis and vaccine challenge studies in monkeys and indicates the potential use of the SFV/SIV monkey model to study the dynamics of SFV and human immunodeficiency virus type 1 (HIV-1) dual infections, recently reported in humans.

While the introductory talk was mainly devoted to simian FVs, the oral presentation by **Magdalena Materniak** (National Veterinary Research Institute, Pulawy, Poland) focused on non-primate FVs. The results confirmed the presence of equine FV (EFV) infections in horses from Poland using virus-specific tools. So far EFV, once reported to be isolated from blood of naturally infected horses, the least known FV, is shown to be the most similar to bovine FV (BFV) [7]. M. Materniak presented development and application of specific diagnostic tools to investigate EFV prevalence among racing and saddle horses, Hucul ponies and Polish Konik horses in Poland. Serological testing based on an ELISA test using recombinant EFV Gag protein revealed that 35% of the serum samples showed reactivity to EFV, while qPCR allowed amplification of EFV *pol* gene sequences in 14.6% of DNA samples. Moreover, when the same sera were tested for antibodies to equine arteritis virus (EAV), equine herpesvirus 1 (EHV 1), and equine influenza virus A1 and A2 (EIV A1, EIV A2), a statistically significant correlation (*p <* 0.03) was found between EFV and EIV A2 infections, while no correlation was noted between EFV and infections with the other viruses. Since the study enrolled almost 300 samples, the results highlight the prevalence of EFV infections of horses.

More data are available regarding infections with BFV. Many reports showed that BFV is present in a high percentage of dairy cattle in different parts of the world [8]. Previous studies reported the presence of BFV DNA in different tissues of naturally and experimentally infected cows without uncovering the primary site of virus replication. **Piotr Kubiś** (National Veterinary Research Institute, Pulawy, Poland) presented the first study that aimed to identify BFV replication sites via BFV RNA detection and quantification in blood and different organs of three experimentally inoculated calves. Using a newly developed one-step RT-qPCR, viral RNA was detected in spleen, lung, liver, parotid and mesenteric lymph nodes and BAL cells of all BFV-inoculated calves while tonsil and submaxillary lymph node samples of only two calves and a single testicle sample were positive. The highest viral RNA copy number was 787 per 1 μg of total cellular RNA in the parotid lymph node of one calf. No viral RNA was detected in thymus, kidney and salivary glands of BFV-inoculated animals or in the PBLs and tissues of control calves. Surprisingly, no viral RNA was detected in saliva samples collected from the BFV inoculated animals, in contrast to the results shown in monkeys, cows and cats naturally infected with SFVs, BFV and feline FV (FFV), respectively. Nevertheless, this was the first report indicating possible replication sites of BFV in cattle based on detection of viral transcripts. 

Another plenary talk was presented by **Florence Buseyne** (Pasteur Institute, Paris, France). She reviewed the cross-species transmission of SFV to humans in Africa and Asia and presented past and current work on humans with zoonotic SFV infection performed in the research unit headed by Antoine Gessain (Pasteur Institute, Unit of Epidemiology and Physiopathology of Oncogenic Viruses, Paris, France). Several reports published so far showed that SFV infection is frequent in persons exposed to nonhuman primates (NHPs) [9]. However, among the 60 relatives of SFV-infected individuals reported in different publications, none was found infected with SFV, clearly suggesting that infected human individuals are dead-end hosts for the virus. F. Buseyne presented the results of studies concerning the presence and the origin of SFVs in human populations at risk for contacts with monkeys and apes in Central Africa. The team collected 2485 blood samples of adults living in villages of South and East Cameroon lowland tropical forest, close to NHP habitats and 300 blood samples of individuals who reported direct contacts (bites, wounds, scratches) with NHPs. In the general population, six persons (0.2%) were found to be infected; in contrast, among hunters and individuals in contact with NHPs, 46 (15%) individuals were found to be infected. These were mostly infected with SFV from apes (37/39, mainly SFV from gorillas) while infections with monkey SFVs were less frequent (2/39). The two risk factors associated with SFV infection in humans were bites and contact with apes. In 11 SFV-infected humans, CD4^+^ and CD8^+^ T lymphocytes and B lymphocytes were the major cellular targets of SFV in peripheral blood [10]. Another study showed that SFV DNA can be detected in PBMCs (13/14, 1–20 copies of SFV DNA/10^5^ cells) and saliva (in 6/14, 1–15 copies of SFV DNA/10^5^ cells) of SFV-infected hunters. In contrast, no SFV RNA was detected in saliva or blood samples. Based on these results and published data, SFV replication in the oral cavity may be more restricted in humans than in NHPs [11]. F. Buseyne concluded that human infection with zoonotic SFV represents a natural and unique model to study the role of viral and immunological factors in restricting viral emergence. Ongoing research was presented in the following oral presentations by two PhD students.

**Caroline Lambert** (Pasteur Institute, Paris, France) discussed the results of her study on examining the presence of neutralizing antibodies in the plasmas of SFV-infected persons living in rural areas in South Cameroon. Neutralization was assessed against three viral isolates from the chimpanzee clade (SFVcpz): SFVcpzPFV and SFVcpzSFV7 strains, isolated from animals, belonging to serotypes 6 and 7, respectively, and SFVcpzBAD327 strain, isolated from a Cameroonian hunter. She showed that plasmas from six SFVcpz-infected persons neutralized either the lab-adapted SFVcpz PFV or both SFVcpzSFV7 and SFVcpzBAD327 isolates. Plasmas from seven uninfected individuals and five persons infected with SFV from monkeys did not neutralize any SFVcpz isolates. Out of the 35 persons infected with SFV from the gorilla clade, plasmas from 21 neutralized SFVcpzPFV only, eight neutralized both SFVcpzSFV7 and SFVcpzBAD327, one neutralized all three strains, and five failed to neutralize any strain. There was a strong correlation in neutralizing titers against SFVcpzBad327 and SFVcpzSFV7 strains (Spearman’s rho = 0.998, *p <* 0.0001), supporting that the zoonotic SFVcpzBad327 strain belonged to serotype 7. Results were also presented showing that SFVs from the chimpanzee and gorilla clades transmitted to humans living in Cameroon share common antigenic properties and belong to at least two serotypes described in non-human primates. Plasmas with no neutralizing activities may belong to persons who failed to raise proper immune response but they might also have been infected with viruses from a distinct serotype. **Léa Richard** (Pasteur Institute, Paris, France) focused her study on the genetic diversity of SFV strains isolated from African Apes and hunters infected with zoonotic strains. Complete or partial SFV *env* sequences, which were amplified from the blood DNA of a series of 30 humans infected with zoonotic SFV, originating mostly from gorilla, and a series of five gorillas or chimpanzees, living in the same area of South Cameroon, revealed that the gorilla strains isolated from the infected individuals segregated into two distinct genetic variants that differed in a 750 bp long fragment of the *env* SU region. Interestingly, this region is similar to the one recently described as a recombinant “hot spot” region in the prototypic SFVmcy-2 strain and the FFV serotypes (see above). One of the variants was present in a captive lowland gorilla and the other in a wild-caught gorilla from Cameroon. She highlighted that preliminary phylogenetic studies suggested that one variant derives from a recombination between a gorilla FV strain and another NHP strain, possibly of chimpanzee origin. 

While zoonotic transmission of SFVs has been already confirmed, information regarding cross-infection of humans by non-primate FVs is still very limited [5,12]. This topic is even more interesting considering that the exposure of humans to non-human primate FVs is restricted to limited human populations, while the exposure to non-primate FVs from companion and life-stock animals is much more likely and possibly affects very large populations. Of note, this includes humans with immature or impaired immune systems, like children or immuno-compromised individuals, whose innate barriers to virus cross-species transmission may be limited [13]; therefore, such groups of humans should be considered as highly exposed to the risk of non-primate FV transmission, especially those having direct contact with cats, cows, and horses. **Magdalena Materniak** presented her preliminary study enrolling serological examination of 117 plasma samples collected from immunosuppressed patients—transplant recipients and 44 control samples—which were tested using recombinant antigens specific for non-primate FVs: Gag and Bet for BFV and FFV, and Gag for EFV. In the group of immunosuppressed patients, two samples showed increased reactivity to BFV antigens, six samples to FFV antigens and eight to EFV antigen, while in the control group, one sample showed higher reactivity to BFV antigen, two to FFV antigens and three to EFV antigen, however PCR did not confirm this data. Due to the questionnaire filled in by blood donors, it was possible to collect some epidemiological data regarding gender, age, place of residence, frequency, and type of contact with cows, horses and cats, and to conduct statistical analyses. Although only a few samples showed over-reactivity to particular antigens, statistical analysis showed a weak positive correlation between net-OD values (the result of ELISA test obtained by subtraction of the background OD from antigen specific OD value of each sample) and frequency of contacts for BFV Gag in immunosuppressed patients. These results show that a zoonotic potential of non-primate FVs cannot be excluded, but more samples should be tested to verify the presented data. 

The session ended with a lecture from **Aris Katzourakis** (Oxford Univeristy, Oxford, UK), who focused on the existing and timely topic of endogenous viral elements in mammalian genomes and the evolutionary history of FVs. In general, retroviruses exist in two forms: endogenous (ERVs), which are inherited through the host germ line, and exogenous, which are horizontally transmitted from host to host. Some retroviruses, like mouse mammary tumor virus (MMTV) or avian leukosis virus (ALV), include both exogenous and endogenous forms. Several ERVs are infectious and can be responsible for animal diseases, but the majority is defective and can be used as genomic ‘fossils’ reflecting the history of past infections of the host. After detecting the first endogenous lentivirus in a mammalian genome [14], A. Katzourakis focused on the evolution of FVs. Since the discovery of endogenous FVs, like SloEFV, which is present in the two-toed sloth (*Choloepus Hoffmannii*), the distribution of FVs has extended to the superorder *Xenarthra*. Taking into consideration the time of genome invasion, it is assumed that SloEFV entered the xenarthan germline about 39 million years ago. This discovery indicated that retroviruses are older than 100 million years, and furthermore, phylogenetic comparison of FVs and their hosts matched and supported coevolutionary history from the Cretaceous until the present day. Moreover, the sequences of three FVs were characterized: (1) the genome from SFV-5, an exogenous FV isolated from *Otolemur crassicaudatus panganiensis* (galago) and renamed PSFVgal [15], showed long open reading frames and a complete viral transcriptome; (2) in contrast, the genome of FV from prosimians, such as aye-aye or lemur (*Daubentonia madagascariensis*; PSFVaye), is endogenous, full of stop codons and other repetitive elements, such as SINEs; (3) while the FV genome from afrotherian Cape golden mole (*Chrysochloris asiatica*; ChrEFV), which was recovered by computational techniques, was found to be highly defective and is thought to be a genuine ancient endogenous virus form. Finally, A. Katzourakis proposed the explanation of the mismatches in FVs and their hosts’ evolutionary history, which can be found in the context of mammalian biogeography. He speculated that about 100 million years ago, eutherians with their FVs colonized the Madagascar-India landmass establishing a stable population, which was separated into two groups about 10 to 20 million years later, when Madagascar and the Indian landmass split. Later the FV variant which colonized the Indian landmass was transported to Laurasia (supercontinent, ancient continental mass in the Northern Hemisphere that included North America, Europe, and Asia (except peninsular India) [16] via continental drift and gave rise to FV in bat (*Rhinolophus affinis*) (RhiFV), while the Madagascar variant initiated the PSFVaye progenitor. Although the ancestral species that introduced FV to lemur and bat lineages and their time of transmission are still unknown [15], A. Katzourakis emphasized that the deep origin of FVs was confirmed and FVs diversified alongside their mammalian hosts all the way back to the base of the diversification of eutherian mammals and at present can be found in all superorders of the *Eutheria* clade (*Xenarthra* (includes sloth), *Afrotheria* (includes golden moles), and *Boreoeutheria* consisting of *Laurasiatheria* (includes bats and carnivores) and *Euarchontoglires* (includes rodents, lagomorphs and primates)) [15].

### 2.2. FV Restriction and Immunity 

FVs are known to be apathogenic in naturally or experimentally infected animals, although they induce persistent, lifelong infections [17]. The most possible route of FVs transmission seems to be saliva, their seroprevalence is high and infections in some captive nonhuman primate species can be 70%–90%. Although FVs establish lifelong persistent infection without evident pathology, the role of cellular factors in FV infections is still not fully understood. So far, the best understood host restriction mechanisms of FVs are Trim5α, which targets FV Gag [18,19] and APOBEC3 (A3), which is counteracted by the FV Bet accessory protein [20,21,22]. There are ongoing studies in different laboratories that are aimed to study already known and still unrevealed mechanisms of FV-host interactions that influence virus replication during infection. In this regard, **Xiaomei Hu** (Nankai University, Tianjin, China) revealed that the N-Myc interactor (Nmi) inhibits prototype FV (PFV) by interfering with the transactivation function of Tas. Overexpression of Nmi reduced PFV replication, whereas its deletion by small interfering RNA increased PFV replication. The Nmi-mediated impairment of PFV replication resulted from the diminished transactivation by PFV Tas of the viral long terminal repeat (LTR) and the internal promoter (IP). Nmi was shown to interact with Tas and abrogate its function by sequestration in the cytoplasm. The results indicate that Nmi may have a role in the host defense against FV infection.

**Cha-Gyun Shin** (Chung-Ang University, Ansung, South Korea) focused in his presentation on transportin 3 (TNPO3), a member of the importin-ß superfamily proteins. Its exact molecular function(s) in retroviral infection is still controversial, despite numerous studies. He discussed the results of his group’s study, which provided evidence for a role of TNPO3 in the replication of PFV. The study revealed that PFV infection was reduced two-fold by knockdown (KD) of TNPO3. The differential cellular localization of PFV integrase (IN) in KD cells compared with control cells pointed to a reduction of viral replication at the nuclear import step. He also found that TNPO3 interacted with PFV IN but not with Gag, suggesting that the IN-TNPO3 interaction is important for nuclear import of the PFV pre-integration complex. 

### 2.3. Virus Replication, Proteins and Assembly

This session was chaired by **Dirk Lindemann** (Technische Universität Dresden, Dresden, Germany) and **Wentao Qiao** (Nankai University, Tianjin, China). **D. Lindemann** opened the session summarizing his personal perspective on FV structural protein biosynthesis and particle egress. His presentation allowed us to learn which particular features of FV are some of the most interesting and motivated him to devote his scientific career to exploring molecular mysteries of these unconventional retroviruses. 

The last three decades of research on FVs revealed their unique replication strategy. The retrovirus taxonomy was updated in 2005 to group FVs into the subfamily *Spumaretrovirinae*, under a single genus *Spumavirus* (the original name for FVs), whereas all other retroviruses are classified in the six genera in the subfamily *Orthoretrovirinae*.

Although the FV RNA genome encodes canonical retroviral structural proteins like Gag, Pol and Env*,* it contains unique features such as an internal promoter in the *env* gene, which drives expression of the accessory genes. Furthermore, the biosynthesis and functions of the FV structural and non-structural proteins bear multiple special characteristics. 

FV Gag is translated, probably by a ribosomal shunt mechanism, as a protein precursor like orthoretroviral Gag proteins. Unlike orthoretroviruses, the proteolytic processing of FV Gag proteins during assembly and release is restricted to a single major cleavage site close the protein C-terminus. Recently, it was shown that Gag precursor cleavage is the initiating event for intra-particle reverse transcription in FV virions. This leads to the presence of nearly full-length double-stranded viral DNA genomes in a significant fraction of released FV virions. These appear to represent the major reservoir of infectious FV particles although some addition reverse transcription was reported to take place upon FV particle entry into host cells. **Martin Löchelt** (German Cancer Research Center, Heidelberg, Germany) cautioned during the discussion of the talk that this may be, however, only the consequence of high intracellular dNTP pools in the tumor cells used to propagate FVs *in vitro*. **D. Lindemann** and **M. Löchelt** agreed that further characterization of virus preparations obtained from primary cell cultures and additional *in vivo* studies on this issue are mandatory to settle this point.

Both the Gag precursor and the large cleavage product constitute the capsid in released FV particles that display a rather immature capsid morphology. The FV Gag proteins lack several canonical motifs found in orthoretroviral Gag proteins, like a major homology region (MHR), Cys-His boxes, or a membrane-targeting domain. Consequence of the latter is the failure of FVs to release virus-like particles (VLP) in the absence of Env coexpression and explains the strictly Env-dependent particle egress strategy. Instead, FV Gag proteins harbor several coiled-coil (CC) domains that are involved in interactions with the viral Env protein, cellular motor protein complexes, or Gag-Gag protein interactions. Furthermore, the Gag C-terminus is extremely rich in glycine and arginine residues. Numerous important functions for FV replications have been attributed to this domain, such as participation in genome and polymerase encapsidation, reverse transcription, capsid assembly and morphology, or chromatin tethering. The controversy regarding the mechanism of Gag nuclear localization was discussed: nuclear localization signals and nuclear export signals within Gag were previously identified that suggested an active import and export of Gag in interphase; however, results from live-cell imaging analysis failed to observe significant import of fluorescent protein tagged Gag into the nucleus of interphase cells. Gag attachment to host cell chromatin in a chromatin tethering signal dependent manner was observed only upon nuclear membrane breakdown during mitosis, which resulted in predominant nuclear localization of the protein in the subsequent interphase.

Oral presentations in the session addressed different aspects of virus replication, synthesis of proteins as well as virus assembly. The first one, presented by **Joris Paris** (CNRS, INSERM, Université Paris Diderot, Paris, France), described the study on nuclear trafficking of PFV, including the disputable topic of Gag nuclear localization, as was mentioned above by **D. Lindemann**. J. Paris showed preliminary results of his work suggesting that PFV Gag might transit through the nucleolus to interact with the viral RNA and to export it from the nucleus. This role of Gag would be reminiscent of the role of Rev in HIV replication. Moreover, he presented the signaling sequences like nuclear export signal (NES), which allows Gag to be exported back to the cytoplasm prior to capsids assembly and virus egress [23], as well as a newly identified nucleolar localization sequence (NoLS) in the C-terminus of PFV Gag, which seems to be necessary and sufficient for nucleolar targeting. To study the nucleolar stage of PFV replication, the investigators used conditions of hypoxia that slows down intracellular trafficking and a molecular trap system to retain Gag into the nucleolus. In both cases, Gag was detected in the nucleolus. To identify a nucleolar passage of PFV RNA, an approach based on a ribozyme fused to a snoRNA (primarily developed for HIV studies) was developed, based upon specific cleavage of PFV RNA in the nucleolus. 

FVs are considered to possess replication characteristics of both orthoretroviruses and hepadnaviruses, but have also some unique features. One of them is the presence of glycine-arginine-rich (GR) sequences at FV Gag C-terminus, grouped in three boxes in PFV, which have been shown to play essential functions in genome reverse transcription, virion infectivity and particle morphogenesis. Some additional functions for RNA packaging and Pol encapsidation were suggested, although the contributions of individual boxes have been controversially discussed. **Martin V. Hamman** (Technische Universität Dresden, Dresden, Germany) presented the study demonstrating that the concurrent deletion of all three PFV Gag GR boxes or the substitution of 23 arginine residues residing in the C-terminal GR box region by alanine abolished both viral and cellular RNA encapsidation (>3000-fold reduced) and in addition also had a negative effect on particle release (3- to 20-fold reduced). Mutants that did not encapsidate Pol were non-infectious. In contrast, deletion of individual GR boxes only had minor effects (two- to four-fold) on viral and cellular RNA encapsidation over a wide range of cellular Gag to viral genome ratios that were examined. Taken together, the study provided the first description of cellular RNA encapsidation into FV particles and characterization of Gag mutants devoid of both viral and cellular RNA. The results suggest that the cooperative action of C-terminal clustered positively charged residues is the main PFV Gag determinant for viral and cellular RNA encapsidation. This indicates that non-primate FVs, which lack clustering of Gag GR sequences in GR boxes, might use a similar mechanism of genome packaging and suggests that nucleic acid binding is a promoting, albeit not essential, mechanism for PFV particle assembly.

Like other retroviruses, PFV interacts with host machineries for successful replication. However, the knowledge of cellular proteins, which directly interacts with PFV structural components in the context of viral replication, is still limited. In the presentation given by **Irena Zurnic** (Technische Universität Dresden, Dresden, Germany), a comprehensive yeast-two-hybrid (Y2H) study was described, which confirmed interaction of PFV Gag with various full-length polo like kinase (Plk) of human and rat origin and identified an essential Plk binding motif in PFV Gag. Furthermore, the investigators determined that functional kinase (KD) and polo-box domain (PBD) of Plks were required for direct interaction with the PFV Gag protein. The functionality of this interaction was determined by assessing replication competent PFV particles bearing Plk-binding motif mutations for transduction efficiency. Hence, I. Zurnic concluded that interaction between PFV Gag and proteins of the Plk family is important for early replication steps of PFV within a host cell. Gag mutants unable to interact with Plk proteins had diminished infectivity, but displayed wild type-like particle release, RNA packaging and reverse transcription efficiency. The exact steps where these particles are impeded in transduction remains to be determined by future analyses.

Since most of the data concerning replication of FVs is based on the studies on PFV, there is a gap in the knowledge on replication events in infections with non-primate FVs, including feline FV (FFV). The presentation of **Martin Löchelt**, who described the studies of his former PhD student **Yang Liu** involving mutagenesis of the N-terminus of FFV Gag, revealed key residues essential for either capsid assembly and/or viral budding via an interaction with FFV Env leader protein (Elp). Importantly, the vast majority of cytosolic FFV Gag is assembled in viral capsids and capsid formation is a prerequisite for genome and Pol incorporation and subsequent Gag processing by the viral protease. The data were discussed relative to a recently developed model for PFV Gag–Elp interactions. The different phenotypic changes of N-terminal Gag mutants, including proteolytic Gag processing, intracellular Gag assembly, and particle budding and infectivity, highlighted their essential, distinct and only partially overlapping roles during viral assembly and budding.

The presentation of **Qiuying Bao** (German Cancer Research Center, Heidelberg, Germany) shed new light on the other non-primate FV, BFV. She presented the molecular characterization of the particle release phenotype of BFV. Importantly, although genomic analyses show close relationship of BFV to other FVs, BFV is tightly cell-associated with an extremely inefficient transmission via cell-free virus particles. In her study Q. Bao investigated the mechanism(s) responsible for the low level of cell-free BFV infectivity, for better understanding the process of viral egress. Variants with enhanced cell-free infectivity were selected by *in vitro* evolution in two separate cultures of BICL (baby hamster kidney cells) and bovine MDBK (Madin-Darby bovine kidney epithelial cells) cells. BFV infectivity increased gradually and plateaued at about 10^6^ FFU/mL for BICl cells and 10^5^ FFU/mL in MDBK cell. Concomitantly, the BFV-specific cytopathic effect (CPE) was lost when increased titers are obtained. The complete *gag* and *env* genes as well as the whole *gag-pol-env* cassette were cloned and sequenced from highly released BFV variants and whereas consistent amino acid changes were detected in both genes, additional changes were apparently cell-type specific or stochastic. Since FV particle budding is Gag- and Env-dependent, cotransfection of the selected genes displayed certain combinations with enhanced budding. Some high-titer clones showed a duplication of a potential budding-relevant late (L) domain and/or the specific and in-frame deletion of terminal integrase sequences from MDBK- and BICl-derived variants. 

**Tiejun Bing** (Nankai University, Tianjin, China) also presented data regarding BFV but focused on transactivation of BFV by the regulatory BTas protein that enhances viral gene transcription and is essential for BFV replication. BTas contains two major functional domains, the N-terminal DNA-binding domain (1–133 aa) and the C-terminal activation domain (198–249 aa), which are necessary for BTas transactivation activity. During construction of BFV full-length genomic DNA clone (pBS-BFV), two clones (pBS-BFV-Y and pBS-BFV-B) with the same restriction enzyme pattern but different replication ability were identified. T. Bing described several chimeric clones and the replication assay, which evidenced the viral C-terminal part was more important for virus replication. Furthermore, transient transfection analyses showed that BTas-B encoded by pBS-BFV-B had higher transactivation ability than BTas-Y encoded by pBS-BFV-Y (~20-fold). Sequence alignment showed that there was only one amino acid polymorphism at position 108 between BTas-B (N108) and BTas-Y (D108) and the functional importance of N108 was confirmed by reverse genetics. In addition, T. Bing indicated that the N108D mutation of BTas did not change its subcellular localization, homodimerization or the ability to activate NF-κB signal pathway, but could obviously enhance its binding ability to viral promoters both *in vitro* and *in vivo*. Interestingly, preliminary results showed that D108N could recover promoter binding and transactivation ability of BTas acetylation-deficient mutant, BTas-K66/109/110R. In addition, the N108D mutation in pBS-BFV-B would reduce its replication ability to the same level as pBS-BFV-Y. Collectively, these findings suggested that N108 directly participates in BTas binding to the viral promoter and it is important for BFV replication.

The session was closed by a talk from **Martin Löchelt** who presented a collaborative study with Bryan Cullen’s lab (Duke University, Durham, USA) on the detection of highly expressed RNA polymerase III-directed retroviral microRNAs of BFV [24]. MicroRNAs (miRNAs) are ~22-nt regulatory RNAs that are expressed by all known multicellular eukaryotes and several of their DNA and RNA viruses. miRNAs exert an additional layer of post-transcriptional control leading to a fine-tuning of cellular and viral gene expression. In several DNA and few RNA viruses, miRNAs are essential, e.g., for maintenance of virus persistence and latency or to promote replication. While numerous viral miRNAs expressed by DNA viruses, especially of the herpes virus family, have been reported, there have been very few reports of miRNAs derived from RNA viruses. The BFV miRNAs described were unusual in that they were initially transcribed by RNA polymerase III as a single, ~122-nt long pri-miRNA with a dumbbell-shaped folding of two imperfect hairpins that was subsequently cleaved to generate two pre-miRNAs to finally yield three distinct, biologically active miRNAs. The three resultant mature miRNAs were found to contribute a remarkable ~70% of all miRNAs expressed in BFV-infected cells. The BFV miRNA cassette is located in the non-coding part of the BFV long terminal repeat “U3” region downstream of the accessory and regulatory *bel* genes. In the discussion, **Axel Rethwilm** (University of Würzburg, Würzburg, Germany) briefly summarized his related findings for SFVagm [25]. These data document the second examples of retroviruses being able to express viral miRNAs using polymerase III promoters embedded in the viral LTRs. The observation that BFV and SFV express viral miRNAs in infected cells adds to emerging evidence that miRNA expression may be a common, albeit clearly not universal, property of retroviruses and suggests that these miRNAs may exert significant effects on viral replication *in vivo*. The data were discussed with respect to FV biology, for instance virus persistence in the host and the very long virus–host coevolution.

### 2.4. FV Vectors

The last session was chaired by **Yahia Chebloune** (PAVAL Lab, University of Grenoble, France) who introduced the audience to the topic of retroviral vectors by providing a brief overview of key information about the structure of retroviruses and the specific features that make them an attractive vector system for genetic transfer. Participants of the meeting became acquainted with sequential steps from avian retroviruses to lentiviruses that guided Y. Chebloune’s group to develop and improve innovative caprine encephalitis arthritis virus (CAEV)-based vectors by constructing a chimeric lentivector (CAEV/SIV/HIV genome) that induced HIV-specific CD8+ T cell responses in both mice and macaque models [26]. A part of the lecture was dedicated to FV vectors. Y. Chebloune pointed out the main difference between other retroviruses and FVs, e.g., that many FV particles contain dsDNA genomes. He mentioned the latest generation of replication-deficient PFV vectors composed of the gene transfer vector and the packaging constructs. Examples of FV vector application included the study on canine leukocyte adhesion deficiency (CLAD) gene therapy and the demonstration of minimal genotoxicity due to FV vector integration in human induced pluripotent stem cells (iPSCs) [27]. In addition, results of studies conducted on FFV-based replication-competent vectors were discussed. Showing the advantages of FV vectors, Y. Chebloune raised the question whether in the context of other retroviral vectors we are ready to move on use of FV vectors. Several recent reports have described successful application of FV vectors for gene therapy protocols providing the demonstration of the proof of concept [28,29], as did the following speakers.

**Janet Lei** (German Cancer Research Center, Heidelberg, Germany) presented a study using FFV vectors as vaccine scaffolds for a long-term immune activation. Using protein sequence alignment against databases of immune epitopes, areas of high homology in FFV proteins that may allow epitope integration without disruption of essential viral functions and virus viability were identified and functionally tested. Here, Bet turned out to be a suited scaffold for epitope insertion. Subsequently, the MHC-I epitope SIINFEKL, derived from ovalbumin, was used as a model antigen. Varying lengths of the epitope and its flanking regions were cloned into Bet in a replication-competent FFV genome and analyzed for proper protein expression, virion formation, replication competence, and long-term genetic stability. The results suggested that some modifications are better tolerated than others. The ability of SIINFEKL-bearing Bet protein mutants and full viruses to present the epitope on the cell surface in the context of MHC-I was examined. In IFN-γ ELISpot assays with anti-Ova cytotoxic T-lymphocytes (CTLs), subgenomic constructs bearing the SIINFEKL epitope significantly activated CTLs and promoted IFN-γ release. A full virus bearing the epitope was also able to activate CTLs, however, to a lower degree. The results indicate that FFV and subgenomic constructs derived from FFV can be used as a T-cell epitope transfer vector for display to antigen-presenting cells.

**Nathan P. Sweeney** (Imperial College London, London, United Kingdom) presented a study that used PFV-based vectors to transduce mesenchymal stem cells (MSCs), a promising adult stem cell for use in regenerative medicine. Transduction efficacy was above 95% demonstrating the applicability of this system for further studies. N. Sweeney proposed and assessed the potential of a novel approach of delivering FV-transduced MSCs directly to murine brains as a therapy for the neurodegenerative lysosomal storage disease metachromatic leukodystrophy.

### 2.5. Discussion on Taxonomy of FVs

A special session was included at the end of the program in the conference for a much-needed discussion on FV taxonomy and nomenclature. This topic was brought up and introduced by Arifa S. Khan, who summarized the history of FV taxonomy from 2005–current, and initiated the discussions by proposing a general format for nomenclature that was similar to other retroviruses such as SIV. This would be particularly useful for designating newly discovered FV isolates. The discussion resulted in several ideas for improving virus nomenclature as well as updating FV taxonomy. Follow-up discussions continued by email exchange and the final proposal will be prepared for publication for broader dissemination and consideration by the scientific community and the International Committee on Taxonomy of Viruses (ICTV). 

### 2.6. Outlook

Inspired by the beautiful setting in Pulawy, we enjoyed a highly interactive meeting with a lot of very meaningful and fruitful discussions and exchanges on all topics of current Foamy virology. We hope that this meeting summary attracts new people to the field and other retrovirologists to the upcoming 11th International Foamy Virus Conference hosted by Antoine Gessain and his team in Paris, France in 2016. 

## 3. Tribute to Axel Rethwilm (1959–2014)

On 29 July 2014, the retrovirology community lost an esteemed colleague and friend. Axel Rethwilm, Chair of the Institute of Virology and Immunology at the Universität Würzburg, Germany, passed away prematurely at age 55. It was a great honor for us to host him at the 10th Foamy Virus Conference just a month earlier, where he attended and actively contributed despite increasing physical impairment due to a heritable, progressive motor neuron disease, which limited his physical abilities over his last years. At the meeting, as always, he was interested, critical and supportive to new ideas and enthusiastic about future projects. We will miss his contributions in coming meetings.

A. Rethwilm was internationally recognized for his seminal contributions on FVs. He was admired by his colleagues and students for his enthusiasm to share his expertise and knowledge about retroviruses and provide valuable insight on FV molecular biology, virology, and gene therapy vectors. He started to work on FVs as a young doctoral student in Dieter Neumann-Haefelin’s lab. Then he joined the Institute for Virus Research at the German Cancer Research Center in Heidelberg, where he was the first to molecularly clone a FV genome, which was originally referred to as human FV (HFV), but later shown to be a chimpanzee FV that had infected a human by cross-species transmission. Regardless, his cloning of an infectious FV molecular clone enabled studies in his and many other laboratories on the biology of FVs that resulted in the identification of unique features and terming FVs as “unconventional retroviruses”. He continued to make significant impact in FV research. 

Axel mentored many students and was successful in transferring his passion about FV research to others, resulting in expanding the small group of FV researchers with a new generation of enthusiastic scientists. His devotion to FVs is exemplified in his recent review alluding to FVs as the most successful of all retroviruses due to their “evolutionary success and genome stability” [30].

## 4. Affiliations

Qiuying Bao, Department of Genome Modifications and Carcinogenesis, German Cancer Research Center, Heidelberg, Germany

Tiejun Bing, Key Laboratory of Molecular Microbiology and Biotechnology (Ministry of Education), Key Laboratory of Microbial Functional Genomics (Tianjin), College of Life Sciences, Nankai University, Tianjin, China

Florence Buseyne, Institut Pasteur, Unité d’Epidémiologie et Physiopathologie des Virus Oncogènes, Institut Pasteur, Paris, France

Yahia Chebloune, Laboratoire Pathogénèse et Vaccination Lentivirales : PAVAL Lab, Université Joseph Fourier Grenoble, France

Antoine Gessain, Institut Pasteur, Unité d’Epidémiologie et Physiopathologie des Virus Oncogènes, Institut Pasteur, Paris, France

Martin Hamann, Institute of Virology, Medical Faculty “Carl Gustav Carus”, Technische Universität Dresden, Dresden, Germany

Xiaomei Hu, Key Laboratory of Molecular Microbiology and Biotechnology (Ministry of Education), Key Laboratory of Microbial Functional Genomics (Tianjin), College of Life Sciences, Nankai University, Tianjin, China

Aris Katzourakis, Department of Zoology, Oxford Univeristy, Oxford, UK

Arifa S. Khan, Laboratory Of Retroviruses, Division of Viral Products, OVRR, CBER, U.S. Food and Drug Administration, Silver Spring, MD, USA

Piotr Kubiś, Department of Biochemistry, National Veterinary Research Institute, Pulawy, Poland

Jacek Kuźmak, Department of Biochemistry, National Veterinary Research Institute, Pulawy, Poland

Caroline Lambert, Institut Pasteur, Unité d’Epidémiologie et Physiopathologie des Virus Oncogènes, Institut Pasteur, Paris, France

Janet Lei, Department of Genome Modifications and Carcinogenesis, German Cancer Research Center, Heidelberg, Germany

Dirk Lindemann, Institute of Virology, Medical Faculty “Carl Gustav Carus”, Technische Universität Dresden, Dresden, Germany

Martin Löchelt, Department of Genome Modifications and Carcinogenesis, German Cancer Research Center, Heidelberg, Germany

Magdalena Materniak, Department of Biochemistry, National Veterinary Research Institute, Pulawy, Poland

Joris Paris, CNRS UMR7212, INSERM U944, Université Paris Diderot, Institut Universitaire d’Hématologie, Paris, France

Wentao Qiao, Key Laboratory of Molecular Microbiology and Biotechnology (Ministry of Education), Key Laboratory of Microbial Functional Genomics (Tianjin), College of Life Sciences, Nankai University, Tianjin, China

Léa Richard, Institut Pasteur, Unité d’Epidémiologie et Physiopathologie des Virus Oncogènes, Institut Pasteur, Paris, France

Marzena Rola-Łuszczak, Department of Biochemistry, National Veterinary Research Institute, Pulawy, Poland

Cha-Gyun Shin, Department of Biotechnology, Chung-Ang University, Ansung, South Korea

Nathan P. Sweeney, Infectious Diseases, Imperial College London, London, United Kingdom

Axel Szabowski, Department of Genome Modifications and Carcinogenesis, German Cancer Research Center, Heidelberg, Germany

Guochao Wei, Department of Genome Modifications and Carcinogenesis, German Cancer Research Center, Heidelberg, Germany

Irena Zurnic, Institute of Virology, Medical Faculty “Carl Gustav Carus”, Technische Universität Dresden, Dresden, Germany
